# Blood-derived ratio indexes associated with severity and prognosis of immune checkpoint inhibitor-related cardiotoxicity: a retrospective analysis

**DOI:** 10.3389/fonc.2025.1676806

**Published:** 2025-10-09

**Authors:** Ling Guo, Zhenli Li, Guangbin Gao, Jing Liu, Zhengkun Guan, Tiezhu Yao, Guang Liu, Qian Jiao, Tenghui Wang, Yansong Wang, Jingtao Ma

**Affiliations:** ^1^ Department of Cardiology, The Fourth Hospital of Hebei Medical University., Shijiazhuang, Hebei, China; ^2^ Department of Radiation Oncology, The Fourth Hospital of Hebei Medical University, Shijiazhuang, Hebei, China; ^3^ Hebei Medical University, Shijiazhuang, Hebei, China; ^4^ Guang’anmen Hospital of Chinese Academy of Chinese Medical Sciences, Beijing, China

**Keywords:** immune checkpoint inhibitor-related cardiotoxicities, systemic inflammatory response index, inflammatory ratios, prognosis, severity

## Abstract

**Background:**

Immune checkpoint inhibitors (ICIs) have transformed cancer therapy but may cause immune checkpoint inhibitor-related cardiotoxicities (iRCs). Blood-derived inflammatory ratios may serve as practical prognostic tools for these life-threatening complications.

**Methods:**

We conducted a retrospective study of 105 iRC patients treated with ICIs between 2020 and 2023. Patients were classified by Common Terminology Criteria for Adverse Events (CTCAE) severity grades. We identified the most important blood-derived ratio indexes at iRC diagnosis associated with the severity of iRCs, 40-day major adverse cardiovascular events (MACEs), and long-term mortality using receiver operating characteristic (ROC) analyses and restricted cubic spline (RCS) curves. Kaplan–Meier survival curves, Cox regression, and subgroup analysis were also performed to evaluate them.

**Results:**

High-grade iRCs patients (n=40) showed a significantly higher system inflammation response index (SIRI) (8.21 vs. 2.21, p<0.001) and neutrophil-to-lymphocyte ratio (NLR) (11.46 vs. 5.81, p=0.001) than low-grade cases. SIRI >3.07 was strongly associated with 40-day MACEs [hazard ratios (HR)=6.56, p<0.001], whereas NLR >7.88 was associated with increased long-term mortality risk (HR = 2.33, p=0.003). Both SIRI and NLR remained significant after adjusting for cardiac biomarkers and clinical variables and were found associated with iRC severity-related cardiac biomarkers.

**Conclusion:**

SIRI and NLR are effectively associated with iRC severity and could stratify the risk of developing poor short- and long-term prognoses. These readily available inflammatory indexes could improve risk assessment and guide clinical decision-making for iRC patients. However, further prospective studies should validate their utility in diverse populations.

## Introduction

Immune checkpoint inhibitors (ICIs) mainly target T-cell activation in order to eradicate tumor cells efficiently (1). ICIs have transformed cancer therapies in recent years. They have demonstrated clear advantages in the immunotherapy of advanced cancers, including renal cell carcinoma, non-small-cell lung cancer (NSCLC), colon cancer, and melanoma (2). Nonetheless, with cancer treatments addressing tumor immune checkpoints advanced, physicians progressively identified immune-related adverse events (irAE). Immune checkpoint inhibitor-related cardiotoxicities (iRCs), the most lethal adverse effect, are the main cause for concern (3).

There have been numerous reports of iRCs, such as myocarditis, cardiomyopathy, pericardial disease, arrhythmia, stable angina or acute coronary syndrome, valvular dysfunction, and stress cardiomyopathy (4). It has been established that cytotoxic T cells, which directly destroy myocytes, fibroblasts, endothelium, and mesothelial cells, may cause damage to the heart. This damage is linked to elevated levels of pro-inflammatory cytokines from activated immune cells (5). Relevant research, however, has demonstrated that a variety of factors influence the exact process behind myocardial T-cell injury (6, 7). Thus, there is still much to learn about the mechanism underlying iRCs (8).

Patients with iRCs have a poor prognosis and would bear heavy financial costs (9). Clinicians should thus identify patients with iRCs who could be more likely to develop a poor clinical outcome. Clinical biomarkers that can predict prognosis and illness severity should be used in conjunction with risk stratification to provide more accurate clinical advice (8). Recently, blood-derived ratio indexes have been explored and showed prognostic value of iRCs for cancer patients receiving ICI therapy. For example, an association between increased neutrophil-to-eosinophil ratio (NER) at iRC onset and the development of severe iRCs was demonstrated (9). In addition, Haj-Yehia et al. have suggested that a neutrophil-to-lymphocyte ratio (NLR) was associated with overall cancer therapy-related cardiovascular toxicity in cancer patients with preexisting cardiovascular disease under ICI therapy (10). However, these studies all have certain limitations, such as a small sample size of 47 and 88 cases, respectively, and inadequate assessment of major adverse cardiovascular event (MACE) occurrence and mortality.

In recent years, many blood-derived ratio indexes have been evaluated for the prognosis of cardiovascular diseases (11, 12). For example, increasing evidence has suggested a link between the system inflammation response index (SIRI) and the prognosis of cardiovascular illnesses such as congestive heart failure and acute coronary syndrome (13–15). Given that patients with iRCs may experience acute coronary syndrome or right heart failure, it is reasonable to hypothesize that part of blood-derived ratio indexes are relevant to the MACEs following the occurrence of iRCs. Moreover, some ratio indexes, such as NLR, are associated with increased long-term all-cause mortality among community-dwelling individuals, which are also associated with the long-term survival outcomes among patients who received the ICI therapy (16–18). However, there is still a lack of systematic analysis to explore the prognostic value of these ratio indexes toward patients with iRCs. As a result, we conducted a retrospective study to evaluate the performance of ratio indexes to affect the short or long-term prognosis of those who were diagnosed with iRCs.

## Materials and methods

### Study design and population

This retrospective study was conducted in the Fourth Hospital of Hebei Medical University (Shijiazhuang, China). Between January 2020 and December 2023, a total of 105 patients diagnosed with iRCs underwent at least one cycle of immune-based therapies in our institution. Inclusion criteria are as follows: (1) patients aged 18 years and older; (2) patients received at least one cycle of ICI monotherapy or immunotherapy in combination with other antitumor treatments; (3) patients diagnosed with iRCs according to the European Society of Cardiology (ESC) Cardio-Oncology 2022 and International Cardio-Oncology Society (IC-OS) guidelines. Consensus statement (19, 20); (4) all iRC cases were confirmed by a senior cardiovascular specialist and an oncologist. Exclusion criteria included the following: (1) patients who could not be followed up; (2) patients who had received other antitumor treatments without undergoing immunotherapy.

The study included 105 patients with iRCs after excluding those who met the exclusion criteria. Patient information included demographics, smoking habits, and medical history including hypertension, diabetes, and hyperlipidemia. Tumor category, stage, the time from the start of immunotherapy to the onset of iRCs (abbreviated as days to iRCs), the time from the diagnosis of iRCs to the occurrence of MACEs within 40 days (abbreviated as days to MACEs), the time from the diagnosis of iRCs to all-cause mortality (abbreviated as days to death), the number of cycles of immunotherapy until iRCs occurred, history of previous tumor treatments, Eastern Cooperative Oncology Group (ECOG PS) performance status, types of ICIs, the values of left ventricular ejection fraction (LVEF), N-terminal pro-brain natriuretic peptide (NT-proBNP), cardiac troponin I (cTnI), myoglobin (Myo), creatine kinase myocardial band (CKMB), lactate dehydrogenase (LDH), SIRI, NLR, systemic immune inflammation index (SII), and platelet-to-lymphocyte ratio (PLR) were recorded and calculated when iRCs occurred. Standard methods were used to measure hematological parameters in the central laboratory of the Fourth Hospital of Hebei Medical University.

### Definition of iRCs and inflammatory hematological markers

The cardiotoxicity caused by ICIs spreads to almost all parts of the heart and includes both inflammatory cardiotoxicity and non-inflammatory cardiotoxicity. The former includes myocarditis, perimyocarditis, pericarditis, and left ventricular dysfunction without myocarditis. The latter includes asymptomatic non-inflammatory left ventricular dysfunction, Takotsubo-like syndrome, coronary vasospasm, arrhythmias, and myocardial infarction. Cardiotoxicities associated with immune checkpoint inhibitors include myocarditis which is the most common cardiotoxic reaction (4, 21). The values used to calculate SIRI, NLR, PLR, and SII formulas were as follows: SIRI = (neutrophil count × monocyte count)/lymphocyte count. NLR = neutrophil count/lymphocyte count; PLR = platelet count/lymphocyte count; SII = (neutrophil count × platelet count)/lymphocyte count.

### Severity stratification strategy

Patients were stratified into a low-grade group and a high-grade group using the Common Terminology Criteria for Adverse Events (CTCAE) version 5.0 criteria published by the US Department of Health and Human Services in 2017. CTCAE grades ≤2 were classified as a low-grade group, and grades ≥3 belonged to the high-grade group.

### Follow-up and outcome

The main focus of this study was to assess the clinical prognosis of the iRC patients, which included the occurrence of MACEs during the 40-day follow-up and all-cause mortality during the long-term follow-up. MACEs are defined as cardiovascular death, cardiac arrest, cardiogenic shock, and hemodynamically significant complete heart block that occurred after the initial diagnosis of iRCs by referencing relevant articles (11, 22). Participants included were followed up for both short-term (40 days after the occurrence of iRCs) and long-term periods. MACE incidence and all-cause mortality data were obtained through observation during hospitalization and subsequent routine telephone follow-up.

### Statistical analysis

Continuous data were evaluated for normality using Shapiro–Wilk tests. Continuous variables were presented as the mean with standard deviation (SD) or the median with interquartile range (IQR) according to whether a variable had a normal distribution, Categorical variables were presented as numbers and proportions (%). Appropriate statistical tests, such as independent-sample t-test, Mann–Whitney U-test, chi-square test, or Fisher’s exact test, were employed to compare the two groups. The univariate logistic regression analysis was used to identify the ratio indexes significantly associated with the grade level of iRCs. Spearman correlation coefficients were utilized to investigate the associations between the ratio indexes and the grade level of iRCs as well as the associations between the ratio indexes and cardiac biomarkers (23). The receiver operating characteristic (ROC) curves of four ratio indexes were utilized to discriminate the occurrence of 40-day MACEs and assisted in finding the ratio index with the highest area under the curve (AUC). The time‐dependent ROC curve analysis was performed to identify the ratio indexes most associated with long-term survival outcomes. The medians of ratio indexes were initially assigned as the cutoff value to discriminate iRC patients into the low- and high-risk groups (24–26). Restricted cubic spline (RCS) curves, with the medians of ratio indexes as the reference values, were then used to assess the relationship between the ratio indexes and hazard ratios (HR) for 40-day MACEs and the long-term survival outcomes. The Kaplan–Meier method was utilized to derive survival curves among the low- and high-risk groups, which were then compared using the log-rank test. The Cox regression analysis was further conducted to confirm the significance of selected ratio indexes associated with clinical prognosis. Subgroup analyses of Kaplan–Meier analysis and Cox regression analysis were also performed to test the efficiency of selected ratio indexes. The level of statistical significance was determined by P < 0.05 (two-sided). R software (version 4.4.1) was utilized for conducting data analysis.

## Results

### Baseline characteristics

This study included 105 individuals diagnosed with iRCs, with a median age of 67 years, of which 74.3% were men. The disparities in the variables between patients experiencing low-grade iRCs and high-grade iRCs are presented in **Table 1**. In contrast to patients who did not experience high-grade iRCs, there were no significant differences in the type of iRCs. Likewise, comorbidities, tumor stage, ECOG PS, type of ICIs, ICI therapeutic cycles, and even previous tumor therapy did not significantly differ between patients with low-grade iRCs and high-grade iRCs. The median interval from initiation of immunotherapy to iRCs and from iRCs to MACEs was respectively 57 days (IQR: 31–98 days) and 4 days (IQR: 2–11 days), but both showed no significant difference between the two groups. However, patients in the high-grade group developed more frequently concomitant irAEs (26.2% vs. 50%; p=0.023) and were associated with all-cause mortality (44.6% vs. 70%; p=0.002). Furthermore, patients who encountered high-grade iRCs demonstrated a significantly shorter duration from iRCs to death compared with those with low-grade iRCs (191.5 days vs. 609 days; P<0.001). Patients who encountered high-grade iRCs demonstrated significantly worse LVEF. In contrast to low-grade iRCs patients, individuals with high-grade iRCs exhibited significantly increased levels of CKMB, Myo, cTnI, and NT-proBNP. Patients with high-grade iRCs had significantly higher SIRI, NLR, and SII values than individuals with low-grade iRCs when iRCs occurred. High-grade iRCs patients showed significantly higher SIRI (8.21 vs. 2.21, p<0.001) and NLR (11.46 vs. 5.81, p=0.001) than low-grade cases.

**Table 1:** Characteristics of enrolled patients.

Characteristics	All participants (all grades) N=105	Low-grade group (grades ≤ 2) N=65	High-grade group (grades≥3) N=40	P value
Basic information				
Age, years	67 [62,71]	67 [63,72]	67 [61.75,71]	0.858
Males, n (%)	78 (74.3)	48 (73.8)	30 (75.0)	1.000
BMI, kg/m^2^	23.0 ± 3.5	23.1 ± 3.4	22.9 ± 3.7	0.731
Smoking, n (%)	46 (43.8)	27 (41.5)	19 (47.5)	0.693
Comorbidities				
Diabetes mellitus, n (%)	34 (32.4)	17 (26.2)	17 (42.5)	0.128
Hyperlipemia, n (%)	36 (34.3)	20 (30.8)	16 (40.0)	0.450
Hypertension, n (%)	41 (39.0)	21 (32.3)	20 (50.0)	0.110
Cardiovascular disease, n (%)	40 (38.1)	23 (35.4)	17 (42.5)	0.602
Stroke, n (%)	14 (13.3)	9 (13.8)	5 (12.5)	1.000
Cancer-related information				
Type of iRCs, n (%)				0.062
Myocarditis, n (%)	55 (52.4)	36 (55.4)	19 (47.5)	
Arrhythmias, n (%)	26 (24.8)	19 (29.2)	7 (17.5)	
Impaired ventricular function, n (%)	20 (19.0)	10 (15.4)	10 (25.0)	
Acute myocardial infarction, n (%)	2 (1.9)	0 (0.0)	2 (5.0)	
Takotsubo-like syndrome, n (%)	1 (1.0)	0 (0.0)	1 (2.5)	
Pericardial diseases, n (%)	1 (1.0)	0 (0.0)	1 (2.5)	
Gastrointestinal tumors, n (%)	58 (55.2)	34 (52.3)	24 (60.0)	0.57
Tumor stage, n (%)				0.526
Ⅰ	1 (1.0)	1 (1.5)	0 (0.0)	
Ⅱ	3 (2.9)	3 (4.6)	0 (0.0)	
Ⅲ	34 (32.4)	22 (33.8)	12 (30.0)	
Ⅳ	67 (63.8)	39 (60.0)	28 (70.0)	
ECOG PS	1 [1, 3]	1 [0, 2]	2 [1, 3]	0.077
Type of ICIs				0.558
PD-1, n (%)	93 (88.6)	59 (90.8)	34 (85.0)	
PD-L1, n (%)	12 (11.4)	6 (9.2)	6 (15.0)	
Prior tumor therapy				
Surgery, n (%)	21 (20.0)	16 (24.6)	5 (12.5)	0.209
Radiotherapy, n (%)	21 (20.0)	10 (15.4)	11 (27.5)	0.209
Chemotherapy, n (%)	82 (78.1)	52 (80.0)	30 (75.0)	0.720
Targeted therapy, n (%)	25 (23.8)	12 (18.5)	13 (32.5)	0.160
ICI therapeutic cycles	2.00 [1.00, 3.00]	2.00[1.00, 3.00]	2.00 [1.00, 3.25]	0.790
Concomitant irAEs, n (%)	37 (35.2)	17 (26.2)	20 (50.0)	0.023*
Days to iRCs	57.00[31.00, 98.00]	57.00[31.00, 98.00]	55.00[31.50, 107.00]	0.966
Days to MACEs	4.00[2.00, 11.00]	15.00[4.75, 28.00]	3.00 [2.00, 7.75]	0.388
Days to death	435.00[148.00, 841.00]	609.00[349.00, 912.00]	191.50[36.20, 380.50]	<0.001*
MACEs, n (%)	30 (28.6)	4 (6.2)	26 (65)	<0.001*
All-cause mortality, n (%)	57 (54.3)	29 (44.6)	28 (70.0)	0.02*
Laboratory data				
LDH	341.00 [231.00, 667.00]	279.00 [205.00, 395.00]	659.00 [358.50, 1015.25]	<0.001*
CKMB, ng/ml	31.50 [11.10, 97.10]	19.20 [9.80, 46.70]	53.70 [21.88, 133.65]	0.005*
Myo, ng/ml	67.00 [43.00, 305.00]	55.00 [35.20, 98.00]	127.45 [58.25, 900.00]	<0.001*
cTnI, ng/ml	0.08 [0.01, 0.37]	0.06 [0.01, 0.16]	0.24 [0.06, 2.00]	<0.001*
NT-proBNP, pg/ml	479.00 [129.00, 2110.00]	172.00 [105.00, 914.00]	1320.00 [432.50, 9003.25]	<0.001*
Echocardiography				
LVEF, %	61.00 [56.00, 64.00]	62.00 [60.00, 65.00]	56.50 [47.00, 63.00]	<0.001*
Inflammatory hematological ratios				
SIRI	3.07 [1.35, 8.78]	2.21 [0.81, 4.31]	8.21 [3.02, 10.80]	<0.001*
NLR	7.88 [3.87, 13.99]	5.81 [3.36, 10.93]	11.46 [5.46, 18.33]	0.001*
SII	1428.98 [805.04, 2523.25]	1142.77 [633.23, 2017.23]	1711.37 [1073.83, 3976.18]	0.003*
PLR	239.39 [158.33, 340.26]	219.80 [138.40, 335.09]	259.20 [214.43, 378.50]	0.09

Data are presented as mean ± standard deviation, median [25th–75th percentile] or number (percentage). *P < 0.05.

BMI, body mass index; ICIs, immune checkpoint inhibitors; PD1, programmed cell death protein 1; PDL, programmed cell death 1 ligand 1; SIRI, systemic inflammatory response index; NLR, neutrophil-to-lymphocyte ratio; SII, systemic immune inflammation index; PLR, platelet-to-lymphocyte ratio.

### Identification of indicators most associated with severity of iRCs patients

**Figure 1:**
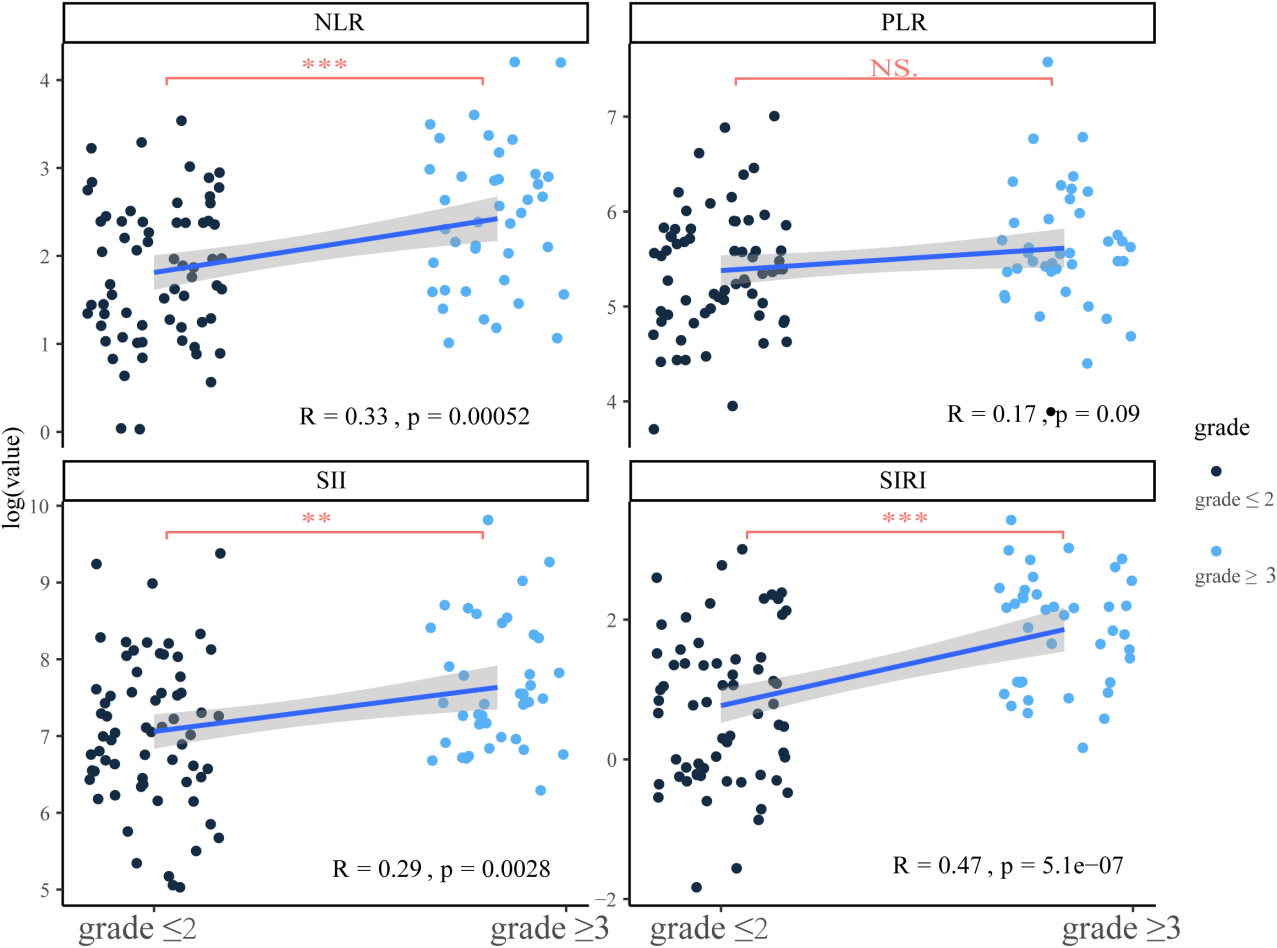
Correlation between ratio indexes at onset and the severity of immune checkpoint inhibitor-related cardiotoxicities and the comparison of ratio indexes in different severity groups. SIRI, systemic inflammatory response index; NLR, neutrophil-to-lymphocyte ratio; SII, systemic immune inflammation index; PLR, platelet-to-lymphocyte ratio; NS, no significance. **P<0.01; ***P<0.001.

In this part, we explored the association between ratio indexes and cardiac biomarkers that may be associated with the severity of iRCs. Initially, significant positive correlations and differences were observed between ratio indexes (NLR, SIRI, SII) and iRC grades, among which SIRI (R = 0.47, P = 0.00052) and NLR (R = 0.33, P = 5.1×10^−7^) showed moderate correlations with iRC grades (**Figure 1**). Meanwhile, the univariate logistic regression analysis (**Table 2**) revealed that a higher NLR [odds ratio (OR)=1.08, 95% confidence interval (CI): 1.03-1.04, P = 0.003] and SIRI (OR = 1.18, 95% CI: 1.08-1.29, P<0.001) at onset of iRCs were significantly associated with an increased risk of developing ≥3 grade iRCs. In contrast, no significant associations were found for SII (P = 0.054) or PLR (P = 0.135). As shown in **Supplementary Figures S2A, B**, SIRI and NLR exhibit relatively significant correlations with LVEF, NT-proBNP, and various serum myocardial enzymology indices including LDH, CKMB, Myo, and cTnI.

**Table 2:** Logistic analysis between iRCs of ≥3 grade and ratio indexes at baseline.

Outcome	Ratio indexes	OR (95% CI)	P value
The occurrence of grades ≥ 3 iRCs	SIRI	1.08 (1.03-1.04)	0.003
	NLR	1.00 (1.00-1.00)	0.054
	SII	1.00 (1.00-1.00)	0.135
	PLR	1.18 (1.08-1.29)	<0.001

iRCs, immune checkpoint inhibitor-related cardiotoxicities; SIRI, systemic inflammatory response index; NLR, neutrophil-to-lymphocyte ratio; SII, systemic immune inflammation index; PLR, platelet-to-lymphocyte ratio; OR, odds ratio.

### Identification of the ratio indexes most associated with prognosis of iRCs patients

**Figure 2:**
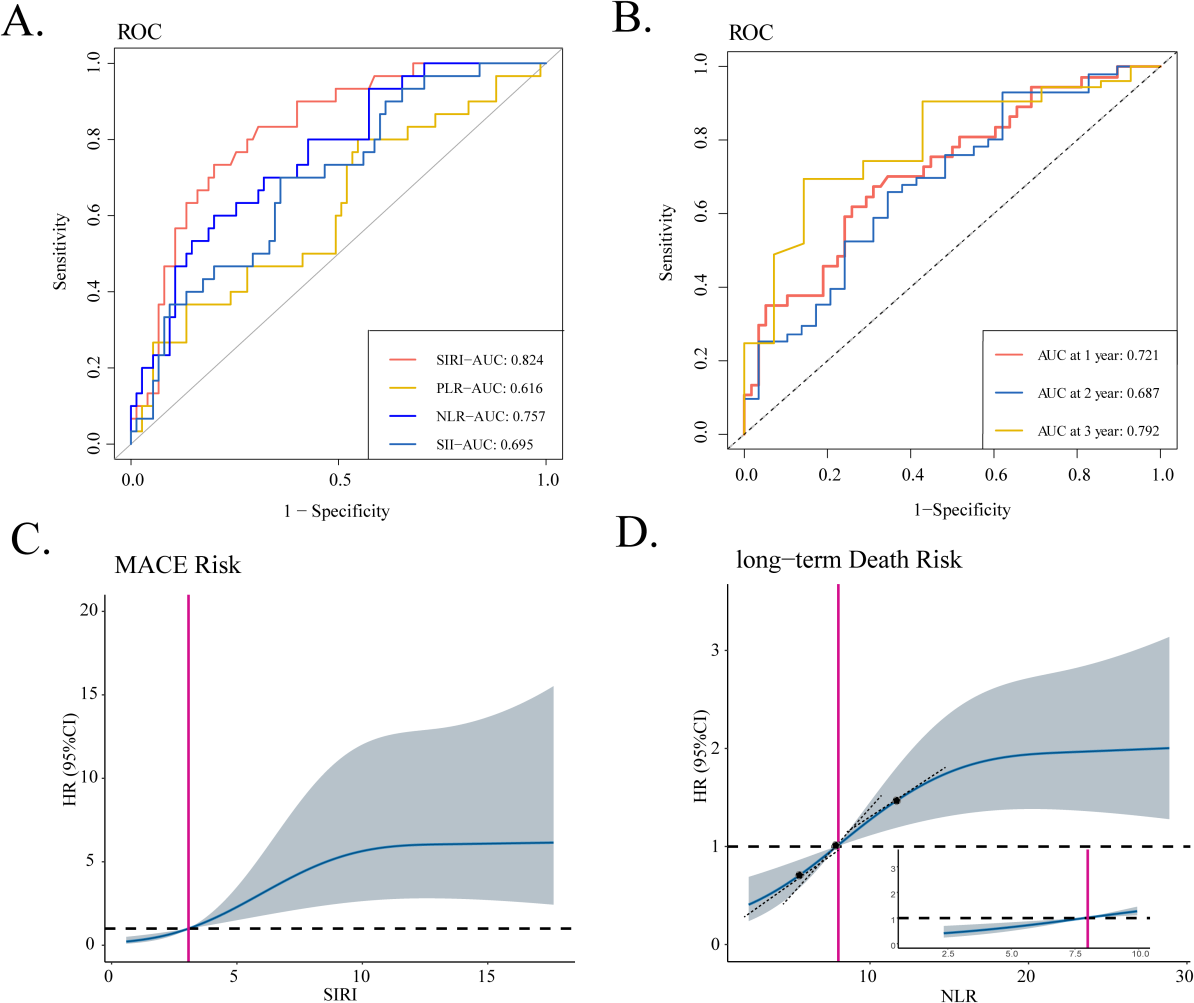
Identification of blood-derived ratio indexes most associated with prognosis of iRC patients. **(A)** The ROC curves of four ratio indexes to discriminate the occurrence of 40-day MACEs. **(B)** The time-dependent ROC curve of NLR to discriminate the all-cause mortality at of 1-, 2-, and 3-year the time points. **(C, D)** RCS curves that show the non-linear relationship between SIRI and hazard ratios of 40-day MACEs as well as relationship between NLR and the hazard ratios of long-term death. iRCs, immune checkpoint inhibitor-related cardiotoxicities; ROC, receiver operating characteristic curve; MACEs, major adverse cardiovascular events; SIRI, systemic inflammatory response index; AUC, area under the curve; NLR, neutrophil-to-lymphocyte ratio; SII, systemic immune inflammation index; PLR, platelet-to-lymphocyte ratio; RCS, restricted cubic spline; HR, hazard ratio.

In the present study, ROC analysis evaluated the ability of different blood-derived ratio indexes to distinguish the occurrence of MACEs at the end of the 40-day follow-up and the occurrence of death at different time points during the follow-up period (**Figures 2A, B**, **Supplementary Figure S1**). Notably, SIRI exhibited the highest AUC value of 0.824 when it comes to the occurrence of short-term MACEs whereas NLR all exhibited the highest AUC value as for the occurrence of long-term death (at the of 1-, 2-, and 3-year time points). We initially selected the median of SIRI/NLR as the cutoff value to discriminate iRC patients into the low- and high-risk groups. Subsequently, when regarding the medians as references, RCS curves were utilized to detect the non-linear relationship between SIRI/NLR and short-term MACEs/long-term death. As **Figures 2C, D** displays, we found a J-shaped association between SIRI and short-term MACEs and an S-shaped association between NLR and long-term death. The RCS curve showed a relatively slow increase of HR by the increase of SIRI before the median and a substantial increase when over the median value (**Figure 2C**). As for the NLR, the RCS curve displayed the highest increasing rate at the point of the median of NLR (**Figure 2D**). Thus, the median value of the onset SIRI/NLR value owned a certain clinical value to serve as the cutoff value in order to identify the high-risk group (> median value) and low-risk group (< median value) for further prognostic analyses.

### Prognostic value of SIRI during the 40-day follow-up

**Figure 3:**
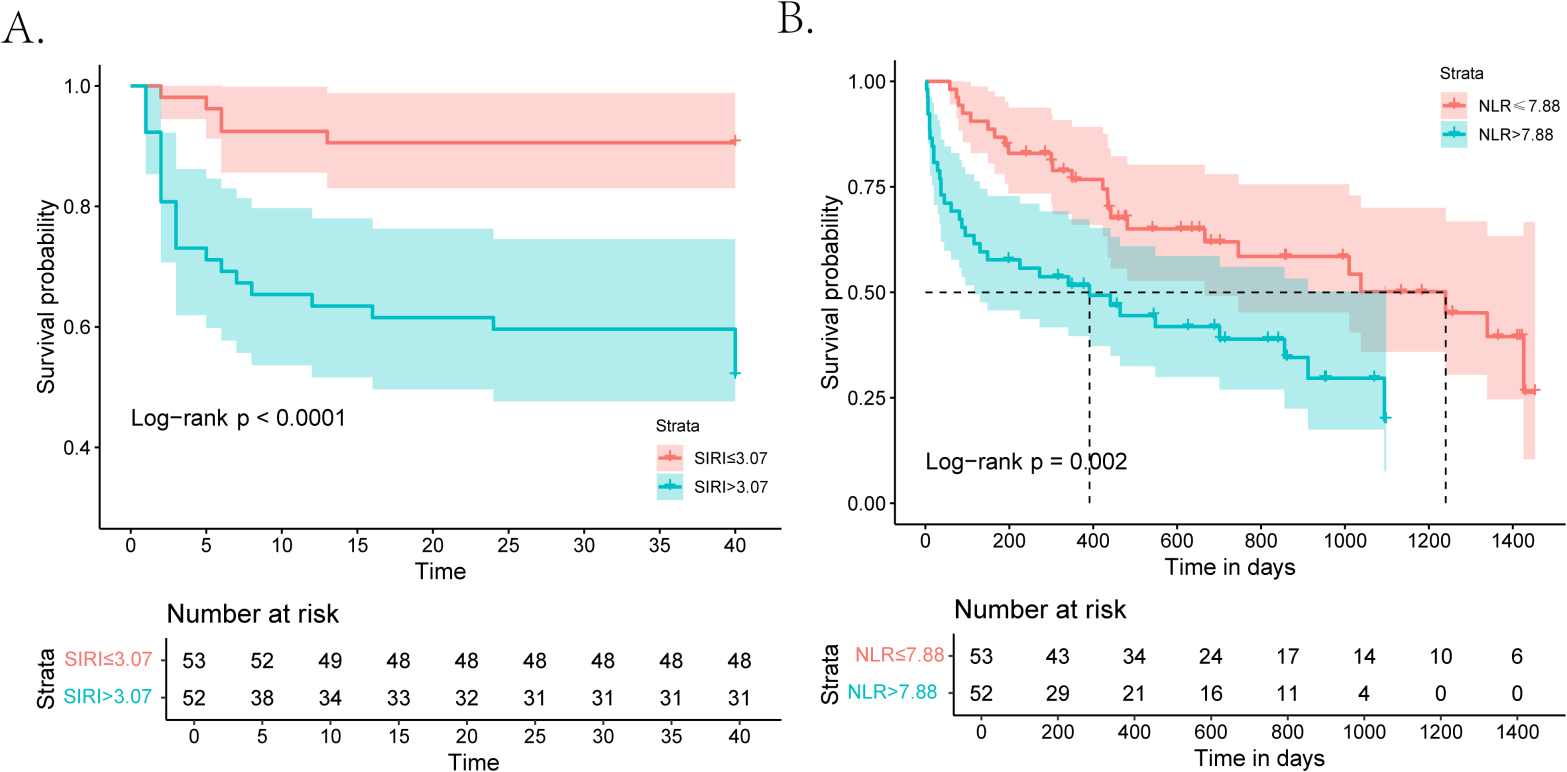
Kaplan–Meier curves for patients with iRCs in terms of 40-day MACEs and long-term mortality. **(A)** Kaplan–Meier analysis of MACEs during the 40-day follow-up in patients with iRCs classified by the median of SIRI. **(B)** Kaplan–Meier analysis of all-cause mortality after a longer follow-up period in patients with iRCs classified by the median of NLR. iRCs, immune checkpoint inhibitor-related cardiotoxicities; MACEs, major adverse cardiovascular events; SIRI, systemic inflammatory response index; NLR, neutrophil-to-lymphocyte ratio.

**Figure 4:**
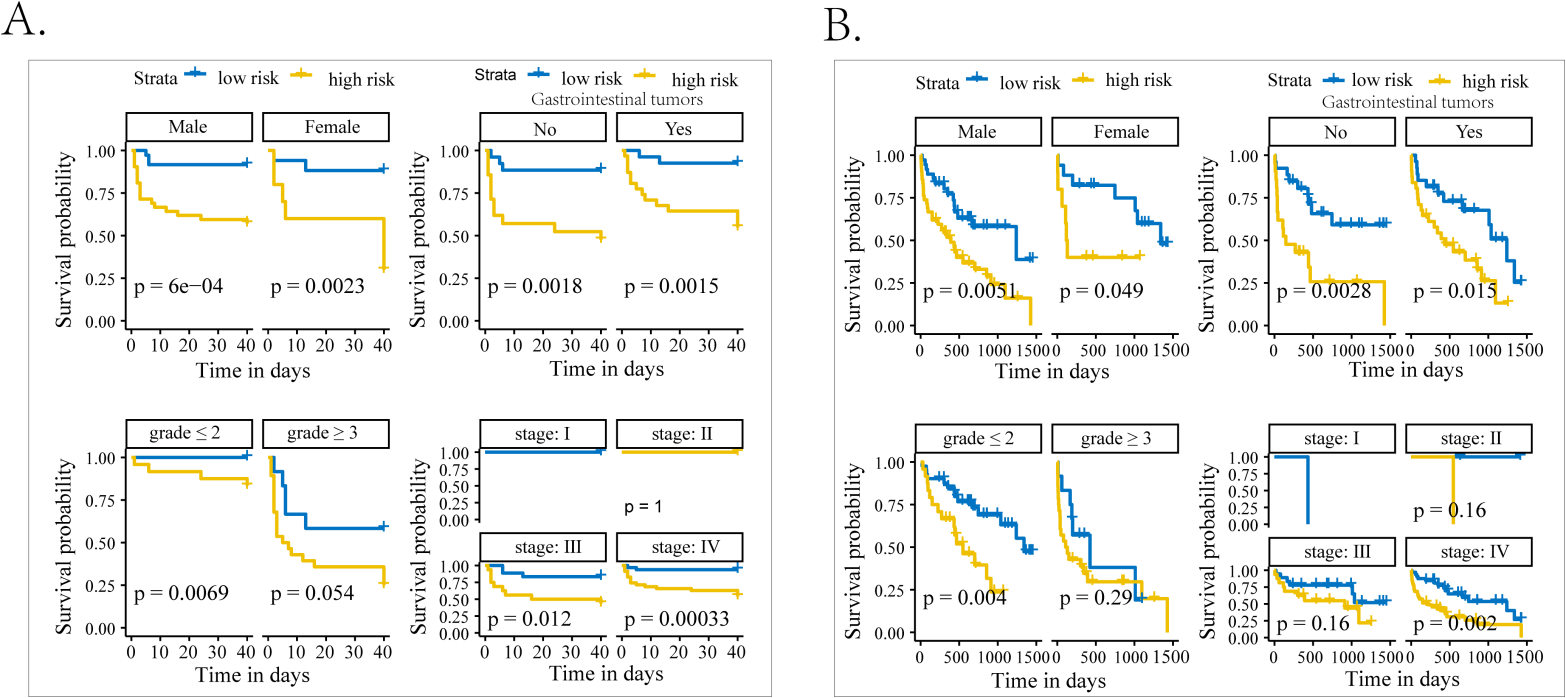
The subgroup analysis of patients with iRCs in terms of Kaplan–Meier analysis. **(A)** Kaplan–Meier curves of occurrence of 40-day MACEs in patients with iRCs classified by the median of SIRI in subgroups with different characteristics. **(B)** Kaplan–Meier curves of occurrence of long-term death in patients with iRCs classified by the median of SIRI in subgroups with different characteristics. iRCs, immune checkpoint inhibitor-related cardiotoxicities; MACEs, major adverse cardiovascular events; SIRI, systemic inflammatory response index.

During the 40-day follow-up period, a total of 30 (28.6%) individuals experienced clinical MACEs. Specifically, there were four patients in the low-grade group and 26 patients in the high-grade group. As displayed in **Figure 3A**, the high-risk group (SIRI > 3.07) exhibited notably high MACE rates and experienced a shorter duration until MACE than the low-risk group (SIRI ≤ 3.07) in the Kaplan–Meier curve. In three COX regression models adjusted by different variables, SIRI all served as a significant risk factor for the occurrence of MACEs in iRC patients whether being analyzed as a continuous or categorical variable (**Table 3**). Furthermore, we performed subgroup analysis to confirm the effectiveness of the above analysis. When stratified by the cancer-related variables, the subgroups divided by SIRI >3.07 also displayed significant prognostic differences in the Kaplan–Meier curves, instead of the cohorts who were in stage Ⅰ−Ⅲ and the high-grade iRC cohort (**Figure 4A**). When performing univariate COX regression models involving only SIRI, the continuous SIRI served as a significant risk factor in different subgroups stratified by different baseline variables, compared with most subgroups for categorical SIRI (**Supplementary Figures S3, S4**).

**Table 3:** Prognostic value of SIRI for 40-day MACEs and NLR for the long-term death among iRC patients.

Outcome	MODEL	SIRI (NLR), continuous		SIRI >3.07 (NLR >7.88)*	
		HR (95% CI)	P value	HR (95% CI)	P value
40-day MACEs	Unadjusted	1.13(1.08-1.19)	<0.001	6.56(2.51-17.15)	<0.001
	Model Ⅰ	1.14(1.08-1.19)	<0.001	6.970(2.64-18.40)	<0.001
	Model Ⅱ	1.11 (1.06-1.17)	<0.001	5.50(2.02-14.93)	<0.001
	Model Ⅲ	1.09 (1.03-1.15)	0.004	6.03(2.05-17.75)	0.001
Long-term death	Unadjusted	1.13 (1.08-1.19)	< 0.001	2.33 (1.34-4.05)	0.003
	Model Ⅰ	1.03 (1.01-1.05)	0.001	2.44 (1.38-4.32)	0.002
	Model Ⅱ	1.02 (1.01-1.04)	0.024	2.20 (1.22-3.96)	0.009
	Model Ⅲ	1.03 (1.01-1.05)	0.007	2.52 (1.34-4.74)	0.004

Model 1: adjusted for age and gender. Model 2: adjusted for variables from model 1 plus LVEF, cTnI, and ln (NT-proBNP). Model 3: adjusted for variables from model 2 plus Myo, CKMB, and LDH. *Reference group in patients with SIRI ≤3.07 (NLR >7.88).

MACEs, major adverse cardiovascular events; SIRI, systemic inflammatory response index; NLR, neutrophil to lymphocyte ratio; HR, hazard ratio.

### Prognostic value of NLR for the long-term follow-up period

The follow-up deadline was June 30, 2024, and the median follow-up was 857 days (IQR: 44–1665 days). As displayed in **Figure 3B**, the high-risk group (NLR > 7.88) exhibited notably high all-cause mortality and experienced a shorter duration until death than the low-risk group (NLR ≤ 7.88) in the Kaplan–Meier curve. In three COX regression models adjusted by different variables, NLR all severed as a significant risk factor for the occurrence of all-cause mortality in iRC patients whether being analyzed as a continuous or categorical variable (**Table 3**). In the subgroup analysis, we further confirmed the effectiveness of the above analysis. When stratified by the cancer-related variables, the subgroups divided by NLR >7.88 also displayed significant prognostic differences in the Kaplan–Meier curves, instead of the cohorts who were in stage Ⅰ−Ⅲ and the high-grade iRC cohort (**Figure 4B**). When performing univariate COX regression models involving only NLR, the continuous NLR served as a significant risk factor in most subgroups stratified by different baseline variables, compared with approximately half of subgroups for continuous NLR (**Supplementary Figures S5, S6**).

## Discussion

### Summary of findings

In this retrospective study, we detected that the SIRI and NLR were associated with the severity of iRCs. As far as is known, there has been no prior investigation into the relationship between SIRI and the iRCs. We found that the SIRI showed a favorable correlation with LVEF, NT-proBNP, LDH, CKMB, Myo, and cTnI, which have been demonstrated to be factors associated with the severity of iRCs (27). It suggests that SIRI and NLR may have the potential in evaluating the severity and prognosis of iRCs like these indicators in clinical practice. Furthermore, our study demonstrated that SIRI functioned as an independent indicator for short-term MACEs, whereas NLR for the long-term all-cause mortality. As a result, SIRI and NLR may be potential biomarkers which own the capacity to transform into a crucial measure for assessing the severity of diseases and the outcomes of clinical prognosis in individuals with iRCs.

### The role of inflammation on iRCs

While the exact development of iRCs was complex and varied, recent studies suggested that inflammation played an important role in the development of iRCs. Although immune checkpoint inhibitors have been used primarily to treat cancer by restoring T lymphocyte attack on malignant cells, activated T lymphocytes had no specificity for tumor cells (28). The mechanism of T-cell damage to the myocardium did not exist only in the direct cytotoxic killing of muscle cells, fibroblasts, endothelial cells, and mesothelial cells by T cells, but also the increased levels of proinflammatory cytokines produced by activated immune cells, leading to bystander damage (29). Furthermore, animal studies have shown that programmed death 1 (PD-1)-deficient mice exhibit increased inflammation, increased serum markers of myocardial damage, and increased infiltration of inflammatory cells [including cluster of differentiation (CD) 8T cells], thereby exacerbating inflammation-induced cardiac damage (7).

### The short-term prognostic value of SIRI for iRC patients

The role of inflammation has been mentioned above. Thus, it was crucial to assess the inflammatory status while evaluating the severity and prognosis of individuals with iRCs. Recently, Li et al. tried to identified the inflammatory blood ratio indexes and demonstrated the prognostic value of NLR, neutrophils-to-high-density lipoprotein cholesterol ratio (NHR), and lactate dehydrogenase-to-albumin ratio (LAR) for the short-term MACE occurrence among patients with ICI-associated myocarditis (ICI-M), which is one of fatal iRCs among immune-therapy patients (11). Moreover, NLR was also identified as an index related to ICI-M severity in their study. In this study, we evaluated and found SIRI as a significant factor associated with the severity and short-term MACE occurrence. By combining NLR and monocyte-to-lymphocyte ratio (MLR), SIRI overcomes the limitations of traditional markers and inflammatory cells in predicting prognosis, which has been proved as an important prognostic factor among patients receiving ICI therapy (30–32). Meanwhile, several studies identified SIRI as a risk factor in predicting MACEs in patients with acute coronary syndromes (33, 34). It was determined by a compound of neutrophils, monocytes, and lymphocytes in the peripheral blood, which have been demonstrated to play a critical role in the pathogenesis of cardiovascular toxicity (35–37). SIRI integrates these three components into a single metric that captures the balance between the aggressive, pro-inflammatory forces (neutrophils and monocytes) and the regulatory, anti-inflammatory forces (lymphocytes). A high SIRI biologically translates to hyperactive innate immunity (elevated neutrophils and monocytes) and suppresses adaptive immunity (depleted lymphocytes). Particularly, Lou et al. revealed that monocytes were significantly involved in the regulation of innate immunity and inflammation associated with ICI-M by performing a single-cell ribonucleic acid (RNA) sequencing analysis (38). Compared with NLR, the involvement of monocytes may better explain the reason why SIRI is a novel inflammation index for iRC patients. Moreover, the positive correlation between the SIRI and cardiac biomarkers suggests that a higher SIRI may reflect a higher cardio-inflammatory load toward patients who received ICI therapy. Therefore, it was logical to conclude that the SIRI is capable of detecting the severity and short-term prognosis of iRCs.

### The long-term prognostic value of NLR for iRC patients

In this study, we found that NLR is also related to the severity of iRCs and owns a long-term prognostic value. In the previous studies, elevated NLR was found to be associated with increased long-term all-cause mortality among community-dwelling individuals with heart failure (HF), which was also associated with the long-term survival outcomes among patients who received the ICI therapy (16–18). As for the long-term prognosis of iRC patients, Liang et al. demonstrated that higher NER (>184.3) correlates with higher mortality in a 2-year follow-up (9). In their study, they found that NLR was significantly elevated among severe iRCs patients. However, they did not further explore the long-term prognostic value of NLR as they have explored NER. As we have mentioned above, NLR was also associated with ICI-M severity. Circulating neutrophils contribute to tumor progression and invasiveness by secreting cytokines, vascular endothelial growth factors, and chemokines, whereas lymphocytes could inhibit the proliferation and metastasis of tumors (16). From this perspective, NLR reflects inflammatory and immune responses in peripheral blood after ICI therapy. A lower NLR probably means a better therapeutic effect of immune therapy and may lead to a better long-term prognostic outcome, which may pay the cost of inducing severer cardiotoxicity. Correspondingly, in the present study, we also identified that a high continuous NLR was positively associated with the cardiac biomarkers at the onset of iRCs, which may reflect the damage load of myocardial cells.

### The advantages of using SIRI and NLR as prognostic indicators for iRCs

As emerging biomarkers, SIRI and NLR could offer numerous advantages for clinical use. Firstly, these two indexes can be initially calculated by the items of routine blood tests derived from peripheral blood sampling, which is convenient, quick, and inexpensive. Furthermore, our study demonstrated that the SIRI and NLR are capable of independently predicting the severity and future prognosis of patients with iRCs. Particularly, it may assist clinicians processing necessary monitoring for iRC patients with a >3.07 SIRI value (for short-term monitoring) or >7.88 NLR value (for long-term monitoring). Therefore, they may have the great potential to become a valuable tool for assessing the future risks faced by individuals with iRCs. For example, patients with SIRI >3.07 at iRC diagnosis should undergo intensified cardiac monitoring [e.g., serial troponin, electrocardiogram (ECG)] during the first 40 days to intercept MACEs. Those with NLR >7.88 warrant long-term surveillance (e.g., echocardiography). Moreover, high SIRI/NLR at the onset of iRCs may prompt earlier immunosuppression (e.g., corticosteroids) or ICI interruption, especially for ICI-related myocarditis, which is fatal and owns a high mortality rate. However, further research is still needed to investigate its potential in evaluating treatment effectiveness and as a treatment target.

### Limitations

There were several limitations to our research. Firstly, this was a single-center, retrospective study. The sample size of the study was limited, and the number of involving factors that could be adjusted is limited. Thus, we propose incorporating SIRI/NLR into iRC management (e.g., risk scores) after collecting enough cases and developing a reliable risk stratification algorithm to personalize monitoring intervals. Secondly, right-censoring existing in our study may introduce potential biases. However, we used standard methods for censored data: both primary statistical methods employed in our study—the Kaplan–Meier estimator for survival probability and the Cox proportional hazards model for multivariate analysis. We also reported the follow-up time using the reverse Kaplan–Meier method. Moreover, outcomes in our study were stratified into short-term (40-day MACEs) and long-term (all-cause mortality) periods, reducing bias from uneven follow-up. Thirdly, during the retrospective follow-up, the causes of death in our study were unable to further distinguish between cardiovascular death and non-cardiovascular deaths such as tumor progression. Further multicenter prospective research is needed to distinguish them for a more specific survival analysis.

## Conclusion

In patients with iRCs, we found that SIRI and NLR are effectively associated with iRC severity and could stratify the risk of developing poor short- and long-term prognoses. These readily available inflammatory indexes could improve risk assessment and guide clinical decision-making for ICI-treated patients. However, further prospective studies should validate their utility in diverse populations.

## Data Availability

The raw data supporting the conclusions of this article will be made available by the authors, without undue reservation.
