# Supplementary material for: Blood-derived ratio indexes associated with severity and prognosis of immune checkpoint inhibitor-related cardiotoxicity: a retrospective analysis
Source: Front Oncol. 2025 Oct 9;15:1676806. doi: 10.3389/fonc.2025.1676806 (PMC12545024; doi:10.3389/fonc.2025.1676806)

Figure Legends of supplementary material

Figure S1 The time-depend ROC curve of SII, PLR and SIRI to discriminate the all-cause mortality at the time points of 1 year, 2year and 3year. ROC, receiver operating characteristic curve; AUC, Area Under the Curve; SII, systemic immune inflammation index; SIRI, Systemic inflammatory response index; PLR, platelet-to-lymphocyte ratio.

Figure S2 The correlation between SIRI/NLR and other cardiac indicators that proved to be associated with the severity of iRCs. SIRI, Systemic inflammatory response index; NLR, neutrophil count to lymphocyte ratio.

Figure S3 Subgroup risk factor analysis of 40-day MACEs by univariate COX regression models involving only continuous SIRI. SIRI, Systemic inflammatory response index.

Figure S4 Subgroup risk factor analysis of 40-day MACEs by univariate COX regression models involving only categorical SIRI. SIRI, Systemic inflammatory response index.

Figure S5 Subgroup risk factor analysis of long-term survival outcomes by univariate COX regression models involving only continuous NLR. NLR, neutrophil count to lymphocyte ratio.

Figure S6 Subgroup risk factor analysis of long-term survival outcomes by univariate COX regression models involving only categorical NLR. NLR, neutrophil count to lymphocyte ratio.

Figure S1

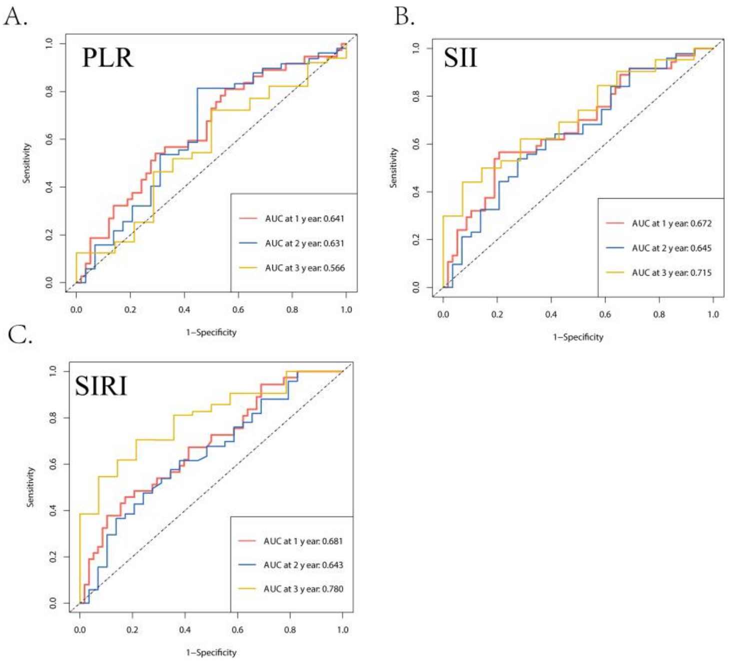

Figure S2

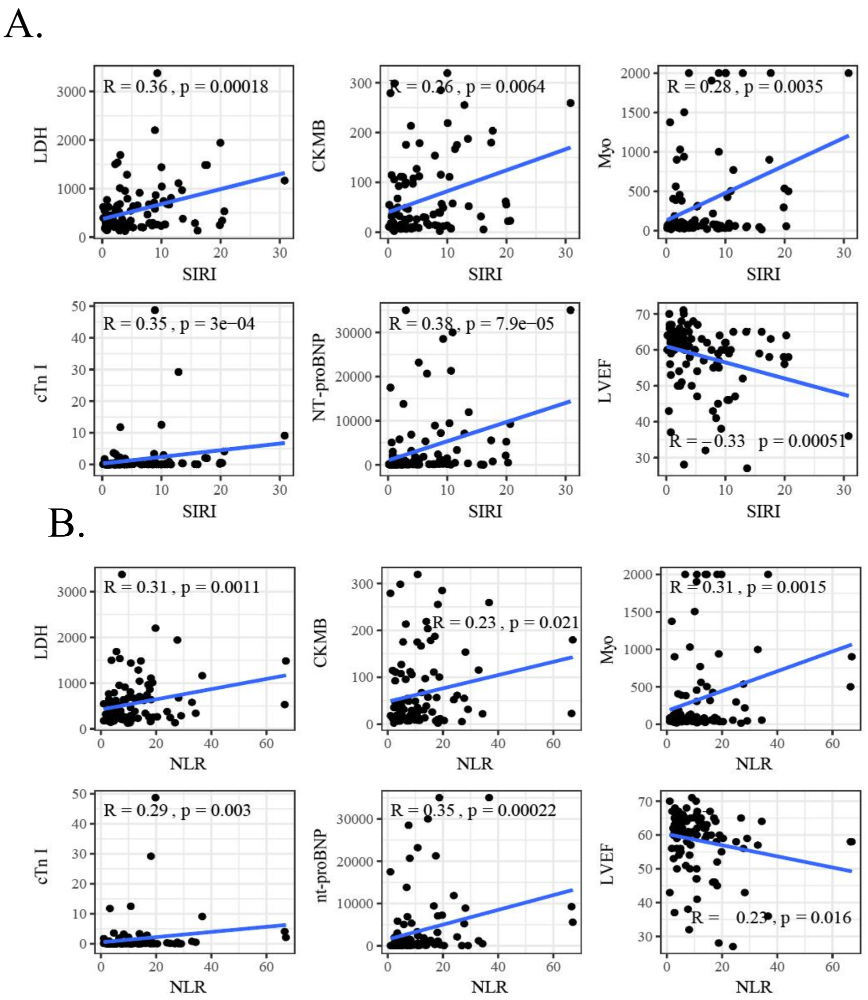

Figure S3

Variable	Count	Percent	hazard ratio	P value
Overall	105	100	1.13	<0.001
sex				
male	78	74.3	1.14	<0.001
female	27	25.7	1.11	0.016
agegroup				
≤65	46	43.8	1.11	0.003
＞65	59	56.2	1.16	<0.001
Diabetes mellitus				
NO	71	67.6	1.14	<0.001
YES	34	32.4	1.12	0.006
hyperlipemia				
NO	69	65.7	1.14	<0.001
YES	36	34.3	1.13	0.011
hypertension				
NO	64	61	1.14	0.002
YES	41	39	1.15	<0.001
surgery				
NO	84	80	1.13	<0.001
YES	21	20	1.17	0.019
preexistingcardiovasculardisease				
NO	65	61.9	1.11	0.001
YES	40	38.1	1.19	<0.001
stroke				
NO	91	86.7	1.14	<0.001
YES	14	13.3	1.13	0.023
concomitantiraes				
NO	68	64.8	1.14	<0.001
YES	37	35.2	1.14	0.002

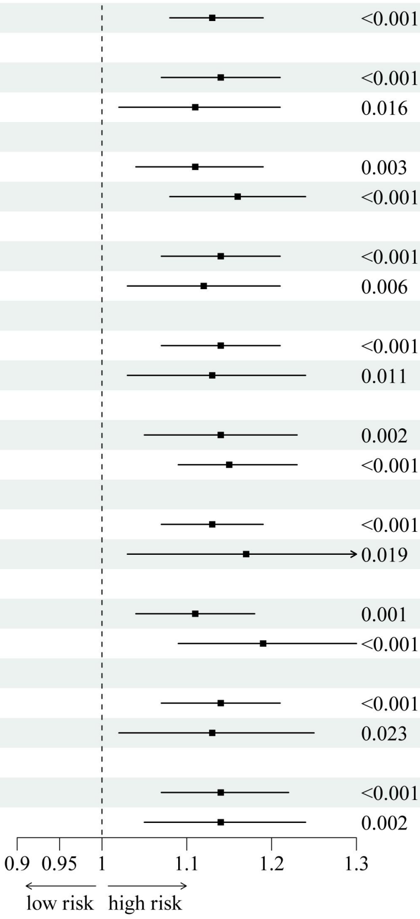

Figure S4

Variable	Count	Percent	Estimate	P value
Overall	105	100	6.56	<0.001
sex				
male	78	74.3	6.46	0.003
female	27	25.7	8.27	0.009
agegroup				
≤65	46	43.8	6.33	0.017
>65	59	56.2	6.68	0.003
Diabetes mellitus				
NO	71	67.6	9.76	0.002
YES	34	32.4	4.26	0.028
hyperlipemia				
NO	69	65.7	7.25	0.002
YES	36	34.3	5.1	0.036
hypertension				
NO	64	61	582220606.47	0.998
YES	41	39	6.29	<0.001
surgery				
NO	84	80	4.78	0.002
YES	21	20	4352835272.02	0.999
preexistingcardiovasculardisease				
NO	65	61.9	8.41	0.001
YES	40	38.1	4.34	0.058
stroke				
NO	91	86.7	6.54	0.001
YES	14	13.3	6.67	0.091
concomitantiraes				
NO	68	64.8	8.89	0.004
YES	37	35.2	4.34	0.022

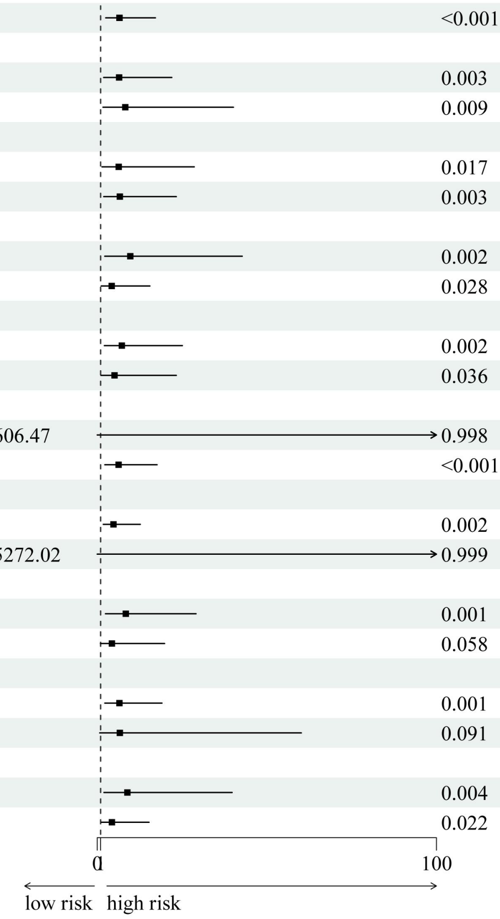

Figure S5

Variable	Count	Percent	Estimate		P value
Overall	105	100	1.03		0.001
sex					
male	78	74.3	1.05		<0.001
female	27	25.7	1.02		0.328
agegroup					
≤65	46	43.8	1.05		0.001
＞65	59	56.2	1.02		0.062
Diabetes mellitus					
NO	71	67.6	1.05		<0.001
YES	34	32.4	1.01		0.376
hyperlipemia					
NO	69	65.7	1.03		0.009
YES	36	34.3	1.04		0.037
hypertension					
NO	64	61	1.02		0.101
YES	41	39	1.14		<0.001
surgery					
NO	84	80	1.05		<0.001
YES	21	20	1.01		0.753
preexistingcardiovasculardisease					
NO	65	61.9	1.02		0.047
YES	40	38.1	1.04		0.007
stroke					
NO	91	86.7	1.02		0.02
YES	14	13.3	1.11		0.008
concomitantiraes					
NO	68	64.8	1.06		<0.001
YES	37	35.2	1		0.836

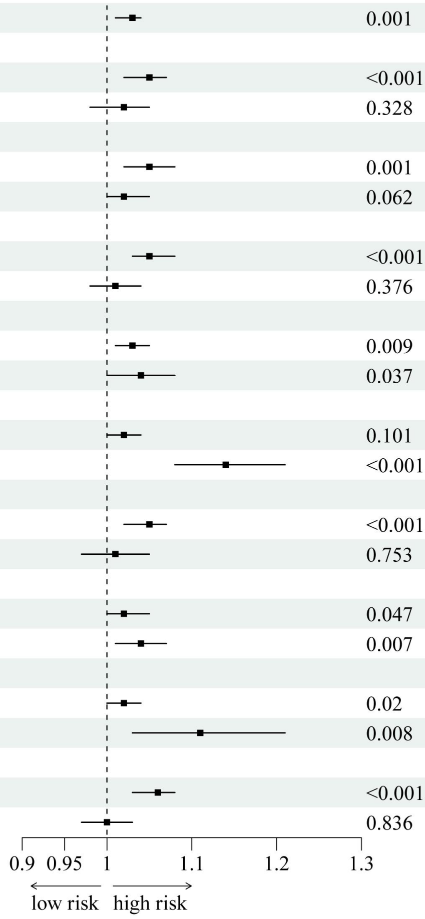

Figure S6

Variable	Count	Percent	Estimate	P value
Overall	105	100	1.13	<0.001
sex				
male	78	74.3	1.14	<0.001
female	27	25.7	1.11	0.016
agegroup				
≤65	46	43.8	1.11	0.003
＞65	59	56.2	1.16	<0.001
Diabetes mellitus				
NO	71	67.6	1.14	<0.001
YES	34	32.4	1.12	0.006
hyperlipemia				
NO	69	65.7	1.14	<0.001
YES	36	34.3	1.13	0.011
hypertension				
NO	64	61	1.14	0.002
YES	41	39	1.15	<0.001
surgery				
NO	84	80	1.13	<0.001
YES	21	20	1.17	0.019
preexistingcardiovasculardisease				
NO	65	61.9	1.11	0.001
YES	40	38.1	1.19	<0.001
stroke				
NO	91	86.7	1.14	<0.001
YES	14	13.3	1.13	0.023
concomitantiraes				
NO	68	64.8	1.14	<0.001
YES	37	35.2	1.14	0.002

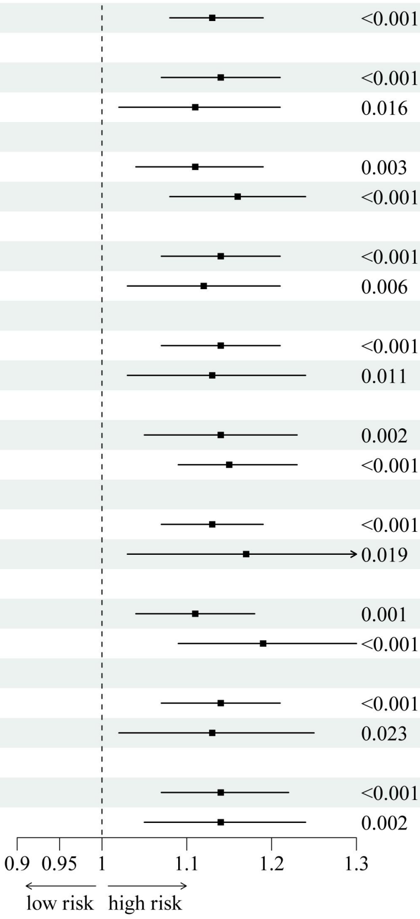

Supplement: Supplementary file 1 [file DataSheet1.pdf]
